# Event-Related Desynchronization and Corticomuscular Coherence Observed During Volitional Swallow by Electroencephalography Recordings in Humans

**DOI:** 10.3389/fnhum.2021.643454

**Published:** 2021-11-26

**Authors:** Satoko Koganemaru, Fumiya Mizuno, Toshimitsu Takahashi, Yuu Takemura, Hiroshi Irisawa, Masao Matsuhashi, Tatsuya Mima, Takashi Mizushima, Kenji Kansaku

**Affiliations:** ^1^Department of Regenerative Systems Neuroscience, Human Brain Research Center, Graduate School of Medicine, Kyoto University, Kyoto, Japan; ^2^Department of Physiology, Dokkyo Medical University, Mibu, Japan; ^3^Division of Rehabilitation Medicine, Dokkyo Medical University Hospital, Mibu, Japan; ^4^Department of Rehabilitation Medicine, Dokkyo Medical University, Mibu, Japan; ^5^Department of Epilepsy, Movement Disorders and Physiology, Graduate School of Medicine, Kyoto University, Kyoto, Japan; ^6^The Graduate School of Core Ethics and Frontier Sciences, Ritsumeikan University, Kyoto, Japan

**Keywords:** swallowing, event-related (de-) synchronization, healthy subject, coherence, electroecephalogram

## Abstract

Swallowing in humans involves many cortical areas although it is partly mediated by a series of brainstem reflexes. Cortical motor commands are sent to muscles during swallow. Previous works using magnetoencephalography showed event-related desynchronization (ERD) during swallow and corticomuscular coherence (CMC) during tongue movements in the bilateral sensorimotor and motor-related areas. However, there have been few analogous works that use electroencephalography (EEG). We investigated the ERD and CMC in the bilateral sensorimotor, premotor, and inferior prefrontal areas during volitional swallow by EEG recordings in 18 healthy human subjects. As a result, we found a significant ERD in the beta frequency band and CMC in the theta, alpha, and beta frequency bands during swallow in those cortical areas. These results suggest that EEG can detect the desynchronized activity and oscillatory interaction between the cortex and pharyngeal muscles in the bilateral sensorimotor, premotor, and inferior prefrontal areas during volitional swallow in humans.

## Introduction

Swallow is a fundamental behavior to maintain life in animals. Although it is partly mediated by reflexive neuronal activities at the brainstem level, multiple areas in the cerebral cortices, such as primary motor and somatosensory cortices and supplemental and premotor cortices, are involved in swallow (Jean, 2001; Ertekin and Aydogdu, 2003; Michou and Hamdy, 2009). Recently, event-related desynchronizations (ERDs) involved in the process of swallow have been reported in bilateral sensorimotor areas using magnetoencephalography (MEG) (Dziewas et al., 2003; Suntrup et al., 2013, 2014, 2015). Generally, ERDs are a decrease of the power in the alpha and beta frequency bands during voluntary movements when compared with that during the rest (nonmovement) (Pfurtscheller and Lopes da Silva, 1999; Li et al., 2018; Chen et al., 2020; Spadone et al., 2020; Xie et al., 2021). They are supposed to reflect cortical activities during voluntary movements (Pfurtscheller and Lopes da Silva, 1999). In the previous works using MEG by the same research group, ERDs were observed in the alpha and beta frequency bands during volitional swallows in the bilateral sensorimotor, premotor, and prefrontal areas (Dziewas et al., 2003; Suntrup et al., 2013, 2014, 2015; Suntrup-Krueger et al., 2018). The emergence of the ERDs detected by their methods was changed in patients with Parkinson’s disease without dysphagia (Suntrup et al., 2013), in patients with functional dysphagia without organic abnormality (Suntrup et al., 2014), in patients with poststroke dysphagia after transcranial direct current stimulation therapy (Suntrup-Krueger et al., 2018), mildly and severely in patients with dysphagic amyotrophic lateral sclerosis, and in healthy subjects after electrical stimulation on the pharynx (Suntrup et al., 2015). There have been few analogous works in electroencephalography (EEG) (Cuellar et al., 2016), whereas EEG is an easier and more economical method for evaluating cortical activities, compared with MEG. In addition to ERDs during voluntary movements, corticomuscular coherence (CMC) has been reported in EEG works using motor tasks of the upper and lower extremities (Mima and Hallett, 1999). CMC is supposed to indicate both corticomotoneuronal activities that projecting the anterior horn cell in the spinal cord (Mima and Hallett, 1999) and ascending sensory feedback from muscles to motor cortex (Witham et al., 2011; Liu et al., 2019). Transcranial magnetic stimulation (TMS) works showed changes in corticomotoneuronal activities by the evaluation of motor-evoked potentials in the pharyngeal muscles in the resting state after repetitive TMS, which increased the cortical excitability of the stimulated area (Michou et al., 2014). In the previous works using MEG, significant CMCs in theta, alpha, and beta frequency bands have been found during tongue protrusion task, which suggests that cortical motor commands can be evaluated in oral apparatus by CMC (Maezawa et al., 2014, 2016; Maezawa, 2017). Although it is important to evaluate the corticomotoneuronal activities during swallow movements with activated submental group muscles and pharyngeal muscles (Ding et al., 2002), there has been no analogous work on them. If ERD and CMC can be detected by EEG recordings using a similar way with the previous MEG studies, it would be easily applicable in clinical practice for the evaluation of swallow dysfunction in neurological patients. Therefore, we investigated them based on the hypothesis that ERDs and CMCs could be measured in the bilateral sensorimotor, premotor, and prefrontal areas as reported by the previous MEG works (Dziewas et al., 2003; Suntrup et al., 2013, 2014, 2015; Maezawa et al., 2014, 2016; Maezawa, 2017). The submental group muscle activity was used to differentiate the swallow activation from the baseline according to the previous MEG works (Dziewas et al., 2003; Suntrup et al., 2013, 2014, 2015) because the onset of the activity was close to the start of the pharyngeal phase (Ding et al., 2002). This work is the observational and descriptive one to investigate the EEG change such as ERD and CMC associated with swallowing.

## Materials and Methods

### Experimental Protocol

#### Participants

Eighteen healthy volunteers (six women and 12 men, mean age 34.2 ± 13.9 years) were recruited for this work. The inclusion criteria were the absence of history of swallow dysfunction and the absence of chronic or acute neurological, psychiatric, or medical diseases. Seventeen subjects were right-handed, and one subject was left-handed according to the Edinburgh Handedness Inventory (Oldfield, 1971). The work protocol was approved by the Committee of Medical Ethics of Dokkyo Medical University, Japan (No. 30008), and written informed consent was obtained from all subjects.

#### Electroencephalography and Electromyogram Recordings

Participants were comfortably seated in an armchair during the recordings. EEG signals were recorded with 32 electrodes. The EEG electrodes, which were the eego™sports active electrodes (ANT Neuro, Netherlands), attached inside the EEG cap were positioned according to the 10–20 international electrode system. The EEG signals were amplified using the eego™sports amplifier. The CPz electrode was selected as the reference electrode. Impedance of all electrodes was <15 kΩ. Data were recorded and saved at a sampling rate of 1 kHz with bandpass filter of the hardware from DC to 260 Hz (ANT Neuro, Netherlands).

Participants were asked to perform volitional swallow without head movements at their own pace with a waiting time of greater than 3 s after 3 mL of water was infused into the oral cavity *via* a flexible plastic tube with 3.3-mm diameter connected with a syringe pressed by an experimenter. The tip of the tube was randomly placed in the left or right corner of the mouth between the buccal part of the teeth and cheek and gently fixed to the skin with tape similar to those in previous MEG works (Suntrup et al., 2014, 2015; Suntrup-Krueger et al., 2018). In the process of the volitional swallow, they were asked to send a small bolus of water deeply to the dorsum of tongue, to have a rest (no tongue movement), and then to perform one time of volitional swallow, and to have a rest (no tongue movement) for a few seconds after the volitional swallow ends. Volitional swallow was repeated for 1 h.

We concurrently recorded surface electromyogram (EMG) with two pairs of bipolar silver electrodes placed on the right and left submental group muscles and unilateral orbicularis oris muscle contralateral to the tube (Okitsu et al., 1998; Nederkoorn et al., 1999; Ding et al., 2002; Vaiman, 2007). The electrodes were connected to a bipolar eego™sports amplifier, and the EMG data were recorded with a sampling rate of 1 kHz and an input range of 150 mV_peak–to–peak_ (ANT Neuro, Netherlands). Swallowing vibrations were recorded with a triple-axis accelerometer (ANT Neuro, Netherlands) which is positioned on the anterior part of the participant’s neck based on the previous works (Suntrup et al., 2014, 2015; Jestrovic et al., 2018; Suntrup-Krueger et al., 2018).

The head movements were monitored by video recordings using two cameras.

### Data Analysis

#### Preprocessing

**FIGURE 1:**
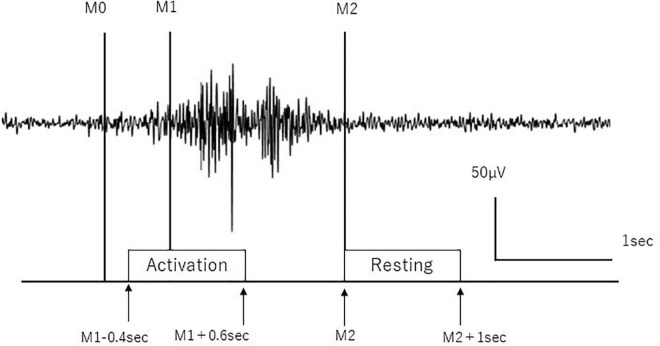
Definition of activation and resting stages. Activation and resting stages are defined according to swallow-related submental group muscle activities. M0 is the time to initiate swallow, M1 is the time to start main muscle activation, and M2 is the time to return to baseline. The surface EMG trace during a single swallow is shown.

We removed the artifacts of the blink and electrooculographic activities from the EEG signals using the independent component analysis (ICA) algorithm (Hyvarinen and Oja, 2000) using EEGLAB MATLAB toolbox (Delorme and Makeig, 2004) (MathWorks Inc., United States). The EEG signals were removed with the power spectral density over 1 × 10^–8^μV^2^/Hz mainly in the frequency domain from 0.01 to 4 Hz as EOG activities (Jung et al., 2000) and with the 50 Hz data as powerline noise in the continuous data from the start to the end of the recording using ICA. EEG signals were segmented based on the onset and offset of separate swallows as determined by EMG signals from the submental group muscles as follows: the beginning of the main muscle activation (M1) and end of the swallow-specific muscle activity (M2) were identified for separate swallows from the submental group muscles. M1 was defined as the time to produce a sustained activity greater than 100% increase in amplitude or frequency of the averaged EMG signal in the resting state. M2 was defined as the time to decrease greater than 50% of the activity in amplitude or frequency of the averaged EMG signal in the resting state according to the previous works (Suntrup et al., 2014, 2015; Suntrup-Krueger et al., 2018). For the analyses of the event-related EEG data, time intervals were defined from –0.4 to 0.6 s in reference to M1 (from “M1–0.4” to “M1+0.6” seconds) as an activation stage and from 2 to 3 s in reference to M2 (from “M2” to “M2+1” seconds) as the resting stage (Figure 1). A third marker (M0) was manually set to distinguish background activity from the onset of swallowing preparation in the EMG to determine mean total swallow duration (from M0 to M2) per subject according to the previous work (Dziewas et al., 2009; Suntrup et al., 2015).

### Electroencephalography Analyses

#### Event-Related Desynchronization

We computed the power spectral density of the denoised EEG data using fast Fourier transform (FFT) for both activation and resting stages. We applied FFT using a boxcar window to 1,000 ms (1,000 points) segments spanning the activation stage (M1 – 0.4 to M1 + 0.6 s), where M1 are the timing of multiple swallowing movements when EMG activity grew larger than the predefined threshold and 1,000 ms segments spanning the resting stage (M2 to M2 + 1.0 second), where M2 is the timing of multiple swallowing movements when EMG activity grew lower than the predefined threshold at each swallowing trial. The FFT data were converted to their absolute value and then averaged across M1 and M2 events. The upper and lower limits of the FFT were 500 and 1 Hz, respectively. The evaluated frequency ranged from 1 to 60 Hz. As we applied FFT to 1,000 ms (1,000 points) segments, 1–60 Hz band was covered by 60 steps using 1 Hz frequency step in the FT computation. We obtained the logarithm of the estimated power spectral densities from 1 to 60 Hz and calculated ERD by subtracting the logarithm of the power spectral densities during the resting stage from that during the activation stage in nine channels (C3, Cz, C4, FC1, FC2, FC5, FC6, CP1, and CP2), which represents the frontal and parietal regions according to the previous works, in which a region of interest was predefined with exclusion of the inferior temporal areas because of tongue movements (Suntrup et al., 2013, 2015). The ERDs of the nine channels were averaged in all subject in the theta (4–7 Hz), alpha (8–14 Hz), and beta (15–25 Hz) frequencies.

To investigate the frequency and temporal properties during swallow, we performed the time-frequency analysis using short-time FT according to the previous works (Kobayashi et al., 2015; Samiee et al., 2015; Usami et al., 2015; Inada et al., 2021). The FT size was 200 points using a boxcar window to 200 ms (200 points) segments and the time shift was 50 ms. The analyzed time period was from 0.5 s before the start of the activation stage {(M1 – 0.4) – 0.5 = M1 – 0.9 s} to 2.5 s after the start of the activation stage {(M1 – 0.4) + 2.5 = M1+2.1 second} and averaged across all the swallow trials. Then, all the subjects’ data of the same nine channels were averaged. The upper and lower limits of the FT were 500 and 5 Hz. The evaluated frequency ranged from 5 to 60 Hz. As the FT size was 200 points with the sampling rate of 1,000 Hz (1,000 points), 5 to 60 Hz band was covered by 12 steps using 5 Hz frequency step in the FT computation.

#### Corticomuscular Coherence

Using the FFT, we computed the cross- and autospectra in the frequency domain of the EEG in each of nine channels (C3, Cz, C4, FC1, FC2, FC5, FC6, CP1, and CP2) and the submental EMG signals segmented for 1 s in the activation stage. The EMG signals were rectified by calculating the root mean square values as the rectified EMG was better to represent the motor unit firing times which reflected the cortical motor inputs (Elble and Randall, 1976; Halliday and Farmer, 2010). The properties of the FFT were the same as those of ERD calculation. The coherence was defined with crossspectra normalized by autospectra in the following equation, in which *f*_*xx*_(*j*), *f*_*yy*_(*j*), and |*f*_*xy*_(*j*)| are the values of auto- and cross-spectra, respectively, at a given frequency *j* (Mima and Hallett, 1999):


$${\left|R_{x\,y}\,\left(j\right)\right|}^{2}=\frac{{\left|f_{x\,y}\,\left(j\right)\right|}^{2}}{f_{x\,x}\,\left(j\right)\cdot f_{y\,y}\,\left(j\right)}.$$

We calculated the coherence and detected the maximal (peak) one in nine channels, each in theta (4–7 Hz), alpha (8–14 Hz), and beta (15–25 Hz) frequency bands.

To investigate the frequency and temporal properties in the CMC during swallow, we performed time-frequency analysis of the CMC using short-time FT in the same way as the ERDs (FT size was 200 points using a boxcar window to 200 ms (200 points) segments and the time shift was 50 ms). The time period was from 0.5 s before the start of the activation stage to 2.5 s after the start of the activation stage. The properties of the short-time FT were the same as those of ERD calculation in time-frequency analysis. We calculated the inverted hyperbolic tangent of the coherence values to make them normally distributed by the following equation:


$$\tanh^{-1}\left|R_{x\,y}\,\left(j\right)\right|=\frac{1}{2}\,\text{ln}\,\left(\frac{1+\left|R_{x\,y}\,\left(j\right)\right|}{1-\left|R_{x\,y}\,\left(j\right)\right|}\right).$$

Then, all the subjects’ data were averaged each in the nine channels, and the grand average of the averaged subjects’ data were calculated over the nine channels.

### Statistical Analysis

As for the ERDs, the averaged ERDs were subjected to one-sample *t*-test (two-sided). The null hypothesis was that the average was zero. As for the CMC, the 95% confidence limit was calculated for the number of trials (*n*) in each subject in the following equation (Mima and Hallett, 1999):


$$C\,o\,n\,f\,i\,d\,e\,n\,c\,e\,l\,i\,m\,i\,t\,\left(95\%\right)=1-{\left(0.05\right)}^{1/\left(n-1\right)}.$$

The existence of CMC was evaluated by the binomial test for each channel in theta, alpha, and beta frequency bands. The null hypothesis was that no subject had a peak of CMC greater than 95% confidence limit in any channel, each in theta, alpha, and beta frequency bands. The Bonferroni correction was used for the multiple comparisons. Effects were considered significant at a *p-*value <0.05. All data were expressed as mean ± SD unless otherwise indicated. The JMP statistical package (JMP Pro 12.2, SAS Institute Inc., United States) was used in each analysis unless otherwise described.

## Results

The numbers of volitional swallows were 206 ± 14.4 times during EEG recordings. The participants’ EMG swallowing parameters were as follows: swallow duration, 1.95 ± 0.69 s and EMG peak-to-peak amplitude, 72.8 ± 36.6 μV. Head movement during EEG recordings was visually inspected. We found the ERDs by EEG recordings similar to the previous works using MEG recordings and CMC during swallow movements.

### Event-Related Desynchronization

**FIGURE 2:**
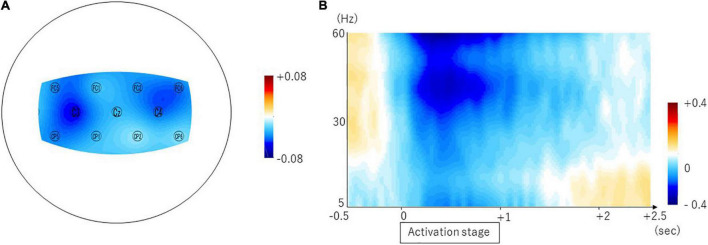
Brain areas showing swallow-related ERDs in the beta frequency band and the temporal property. The topomap display of the swallow-related ERDs is shown in panel **(A)**. The ERDs are prominent in the bilateral sensorimotor cortices. The temporal property is shown in panel **(B)**.

We found the ERD in the frontal and parietal areas in the beta frequency band (Figure 2A). The averaged ERDs for the nine channels (C3, Cz, C4, FC1, FC2, FC5, FC6, CP1, and CP2) were significantly different with zero in the beta frequency band (–0.034 ± 0.048, 95% CI [–0.05 –0.01], *p* = 0.0074) but not in the theta (0.006 ± 0.067, 95% CI [–0.028 0.039], *p* = 0.7190) and alpha (0.017 ± 0.041, 95% CI [–0.003 0.038], *p* = 0.0917) frequency bands (Figure 2A). The Table 1A shows the difference calculated by subtracting the logarithm of the power spectral densities during the resting stage from that during the activation stage (ERD or event-related synchronization (ERS)) in the 31 channels. The Figure 2B shows the temporal modulation of the averaged ERD values of the nine channels during the time period from 0.5 s before the start of the activation stage to 2.5 s after the start of the activation stage. The ERDs emerged immediately before the activation stage and maintained during the activation stage.

**TABLE 1:** The ERD or ERS (mean ± SD) is calculated by subtracting the logarithm of the power spectral densities during the resting stage from that during the activation stage in all channels in Table A.

(A)					
EEG channel	ERD/ERS	EEG channel	ERD/ERS	EEG channel	ERD/ERS
Fp1	−0.002 ± 0.062	FC6	−0.008 ± 0.080	CP6	−0.050 ± 0.077
Fpz	−0.010 ± 0.067	M1	0.013 ± 0.061	P7	−0.013 ± 0.061
Fp2	−0.004 ± 0.069	T7	−0.018 ± 0.066	P3	−0.026 ± 0.062
F7	0.009 ± 0.071	C3	−0.043 ± 0.069	Pz	−0.015 ± 0.056
F3	−0.013 ± 0.053	Cz	−0.026 ± 0.054	P4	−0.038 ± 0.071
Fz	−0.025 ± 0.048	C4	−0.044 ± 0.060	P8	−0.022 ± 0.063
F4	−0.024 ± 0.064	T8	−0.041 ± 0.072	POz	0.003 ± 0.037
F8	0.011 ± 0.098	M2	0.017 ± 0.074	O1	0.008 ± 0.078
FC5	−0.009 ± 0.069	CP5	−0.039 ± 0.065	Oz	0.009 ± 0.077
FC1	−0.025 ± 0.050	CP1	−0.033 ± 0.045		
FC2	−0.030 ± 0.043	CP2	−0.051 ± 0.049		

**(B)**			

**EEG channel**	**Theta frequency band**		**Alpha frequency band**		**Beta frequency band**	

Fp1	0.28		0.61		0.56	
Fpz	0.67		0.67		0.61	
Fp2	0.39		0.72		0.72	
F7	0.39		0.72		0.50	
F3	0.39		0.72		0.67	
Fz	0.39		0.50		0.72	
F4	0.39		0.61		0.78	
F8	0.39		0.61		0.50	
FC5	0.44		0.72		0.50	
FC1	0.33		0.61		0.72	
FC2	0.44		0.78		0.67	
FC6	0.50		0.78		0.72	
M1	0.44		0.61		0.78	
T7	0.39		0.56		0.56	
C3	0.39		0.39		0.61	
Cz	0.33		0.61		0.78	
C4	0.61		0.61		0.44	
T8	0.44		0.56		0.44	
M2	0.56		0.72		0.61	
CP5	0.33		0.50		0.39	
CP1	0.39		0.67		0.89	
CP2	0.44		0.39		0.50	
CP6	0.50		0.61		0.44	
P7	0.56		0.78		0.56	
P3	0.50		0.50		0.50	
Pz	0.50		0.72		0.83	
P4	0.61		0.67		0.50	
P8	0.44		0.67		0.39	
POz	0.72		0.72		0.67	
O1	0.72		0.67		0.61	
Oz	0.44		0.50		0.39	

*n = 18, mean ± SD.*

*The ratio of the subjects with a significant coherence in all 18 subjects is presented in Table B. n = 18.*

### Corticomuscular Coherence

**FIGURE 3:**
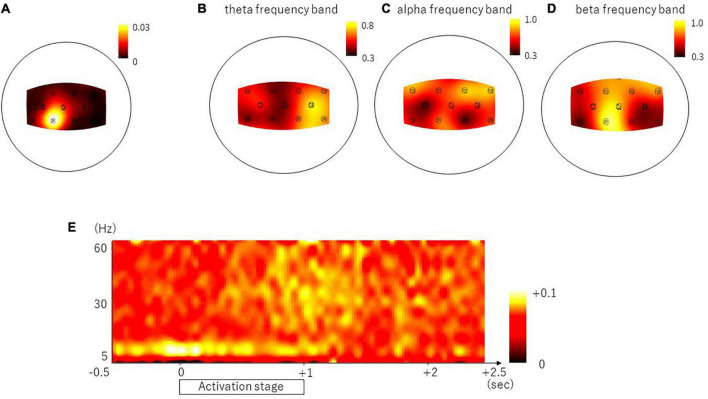
Brain areas showing the representative result of the CMC, the ratio of subjects with significant coherence and the temporal property. Panel **(A)** shows the topomap display of the CMC in the representative subject whose peak value was found within beta frequency band (16 Hz). Panel **(B–D)** shows the topomap displays of the ratio of the subjects with significant coherence in the theta frequency band **(B)**, alpha frequency band **(C)**, and beta frequency band **(D)**. The temporal property is shown in panel **(E)**.

The representative result of the CMC is shown in Figure 3A. In the frontal and parietal areas, we found significant CMC in theta, alpha, and beta frequency bands in all nine channels (theta frequency band, C3, C4, FC2, FC5, FC6, and CP2, *p* < 0.0001, and FC1, Cz, and CP1, *p* = 0.0010; alpha frequency band, Cz, C4, FC1, FC2, FC5, FC6, and CP1, *p* < 0.0001, and C3 and CP2, *p* = 0.0018; and beta frequency band, C3, Cz, FC1, FC2, FC5, FC6, CP1, and CP2, *p* < 0.0001, and C4, *p* = 0.0002). The ratio of the subjects who had the peak of CMC greater than 95% confidence limit was represented in Table 1B and in the topographical mapping in Figures 3B–D. The Figure 3E shows the temporal modulation of the averaged CMC values of the nine channels. The CMC in relatively low-frequency band including alpha frequency band emerged at the early phase of the activation stage, whereas the CMC in relatively high-frequency band including beta frequency band emerged at the late phase of the activation stage.

## Discussion

We found the ERDs during the volitional swallow by EEG recordings in the bilateral sensorimotor, premotor, and prefrontal areas in a consistent way as previously reported in MEG works. Furthermore, we found the significant CMC in those areas during the volitional swallow movement.

Event-related desynchronizations in the beta frequency band were most evident in C3 and C4, which corresponds to the middle precentral gyrus in this work. The somatotopy for pharyngeal muscles is arranged in a more medial part than that for tongue muscles (Hamdy et al., 1996; Dziewas et al., 2003). The previous MEG work reported the ERDs during volitional swallow in more medial parts, compared with the ERDs during tongue movement (Dziewas et al., 2003), whereas the previous TMS work showed that pharyngeal muscles were arranged more medial and oral muscles were more lateral in the cortical representations (Hamdy et al., 1996). Our findings indicate cortical activities related to pharyngeal muscles during swallow.

The other brain imaging works also showed swallow-related cortical areas (Kern et al., 2001; Martin et al., 2001; Kober et al., 2015; Kober and Wood, 2018). The bilateral primary sensorimotor cortices, insula, cingulate, and parietal regions were represented during volitional swallow in the fMRI works (Kern et al., 2001; Martin et al., 2001). Although the activity of deep brain structure is difficult to measure with the EEG recordings, it may have influenced the observed sensorimotor activities by the EEG recordings. The previous fNIRS works showed the increased blood flow of the bilateral inferior frontal gyri (Kober et al., 2015; Kober and Wood, 2018), which were included in the analyzed areas for the ERDs in this work.

Event-related desynchronizations in the sensorimotor area are associated with the neuronal activities in not only the production of appropriate motor outputs but also in the processing of somatosensory information from the moving muscle (Pfurtscheller and Lopes da Silva, 1999). Processing of oropharyngeal sensation and complex motor execution are essential during swallow to prevent aspiration (Dziewas et al., 2003; Ertekin and Aydogdu, 2003). Therefore, alteration in ERDs can occur according to the dysphagia symptoms and recovery process (Suntrup et al., 2014; Suntrup-Krueger et al., 2018).

In the process of swallow, the tongue movement is important. To transit a bolus of water posteriorly at the oral phase, the apex of the tongue elevates and contacts with the hard palate and the bolus is propelled posteriorly in the oral cavity. At the pharyngeal phase, the tongue is retracted posteriorly to keep the oropharynx closed at the same time with the elevation and closure of the larynx. After the bolus transits pharynx, the tongue is relaxed and returned to the resting position (Logemann, 1988). In the previous work using the ROIs that covering the lateral areas from the C3 and C4, the tongue thrust execution induced the alpha ERD in the left hemisphere whether the tongue thrust left or right, and beta band ERD in the left hemisphere when the tongue thrust right, suggesting the left-sided dominance in the tongue movement (Sakihara and Inagaki, 2015). In our work, beta band ERD during the swallow was induced in the bilateral sensorimotor cortices. As the tongue movement is not lateralized during swallow, the side-dominance may not have been found. In the other previous work, ERD, ERS, or nothing was induced during the press of the tongue on the roof of the mouth in the primary motor cortex (Morash et al., 2008). The ERD may depend on how the tongue moves. In the previous MEG work that using the tongue electrical sensory stimulation, 20-Hz ERD was shown around 400 ms after the stimulation in the bilateral temporoparietal areas. The sensory inputs to the tongue also induced the beta band ERD. In our work, the ERD during swallow was induced in more medial and rostral parts probably because it was mixed with motor efferents and sensory afferents to the tongue and pharynx. Swallow movement itself requires various muscle activities in the tongue and the pharynx. It is difficult to differentiate specific brain activities for a specific muscle because complex activities of various muscles are necessary for swallow movement. The ERDs found in this work are brain activities during swallow including tongue and pharyngeal movements.

Event-related desynchronizations are detected by comparison with brain activity during a specific event with that during baseline (nonevent) period in the event-related design, instead of comparison with a control task. ERDs have been reported in motor and cognitive tasks without control experiments (Pfurtscheller and Lopes da Silva, 1999; Li et al., 2018; Spadone et al., 2020; Xie et al., 2021). According to the previous works, we detected the brain activity during swallow movements by subtracting that during rest. The ERDs in work emerged at the immediately preceding time of the swallow movements and disappeared around the end time of the swallow movements (the end of the submental EMG activities). The ERDs showed prior to movement onset over the contralateral sensorimotor region and ended after the motor execution in the hand and foot movements in the previous report (Pfurtscheller and Lopes da Silva, 1999). The temporal property of the ERDs during the swallow was similar with the previous finding.

We did not investigate relationships between the ERD and swallow movements because they are beyond the scope of this EEG descriptive work. It is the limitation of this work. The next step would be necessary to investigate relationships between the ERD and swallow movements using correlation, decoding, or validation methods.

We have found that the significant CMC existed in all nine channels covering the lateral and medial parts of the sensorimotor area in theta, alpha, and beta frequency bands in accordance with the previous works (Maezawa et al., 2014, 2016; Maezawa, 2017). For the finger muscles, CMC was not consistently observed in the theta and alpha frequency bands (Mima and Hallett, 1999). However, the previous MEG works reported the CMC in the beta band at 15–35 Hz and the low-frequency band at 2–10 Hz and its contralateral side-dominance in both sides of the tongue during the 2-min tongue protrusion mainly contracting genioglossus muscle (Maezawa et al., 2014, 2016). The CMC in the low-frequency band was suggested to be oscillatory proprioceptive feedback from the tongue muscles to the primary sensory cortex (Maezawa et al., 2016). Swallow consists of a complex series of the pharyngeal and tongue movements. The CMC during swallow may have been partly produced by tongue motion because the submental group muscles included the suprahyoid muscles attached to the hyoid although the muscles working during the swallow are different from those during the tongue protrusion.

As for the temporal property of the CMC, the CMC in low-frequency band was dominant in the early swallow stage with low activity of the submental group muscles and the CMC in beta band frequency was shown with the high activity of the submental group muscle in the late part of the swallow. Previous works showed that the CMC in the beta band frequency reflected the cortical motor commands during a steady tonic muscle contraction and that it did not appear during the initial parts of the movement before the steady contraction (Kilner et al., 1999; Mima and Hallett, 1999). It suggests that the CMC in the beta band may indicate the neural state during a few hundred milliseconds after the pharyngeal movements reached the maximum, that is, the peak of laryngeal elevation. Moreover, it may reflect sensory afferents to motor related areas (Baker et al., 2006). Therefore, the CMC in the beta band frequency might reflect both of motor commands and of sensory afferents in the late part of the swallow. The CMC in the low-frequency band was shown in the early part of the swallow. The previous MEG works showed that the CMC in the low-frequency band reflected the sensory feedback to M1 area with a delay of about 80 ms after CMC in the beta frequency band. However, the CMC in the low-frequency band was only shown without the CMC in the beta frequency band in the early part of the swallow. It might suggest that different networks activate in the early part and in the late part of the swallow. Swallow is a complex motion with multiple muscles working at various timings. Therefore, it is difficult to differentiate precisely the timing of cortical commands with that of the muscle contraction unlike previous MEG works. A future work would be necessary to reveal it.

Corticomuscular coherence does not inform us of the directionality of signals between the EEG and the EMG (Liu et al., 2019). Although it is beyond the scope of this EEG descriptive work, the next step would be to investigate the directionality using causality analyses such as Granger causality analysis (Witham et al., 2011).

The relationship between the submental EMG activities and the phases of the swallow movement was reported by using electroglottography (EGG) in the previous work (Nederkoorn et al., 1999; Ding et al., 2002). Although the submental EMG activities indicate the final oral and pharyngeal phases, it is difficult to differentiate the end of the oral phase from the start of the pharyngeal phase only by the EMG activity. In future, the relationship between swallow phases and EEG activity is to be revealed with the concurrent EEG and EGG recordings.

In conclusion, this work reported that EEG recordings with a small number of electrodes can detect ERDs in the bilateral sensorimotor cortices and oscillatory interaction between the cortex and pharyngeal muscles during volitional swallow in humans. The EEG is an easy and economical equipment for the clinical use compared with MEG, which is available in community hospitals. It might be a useful technology for the evaluation of cortical function during swallow in both healthy subjects and patients with dysphagia.

## Data Availability Statement

The raw data supporting the conclusions of this article will be made available by the authors, without undue reservation.

## Ethics Statement

The studies involving human participants were reviewed and approved by the Committee of Medical Ethics of Dokkyo Medical University. The participants provided their written informed consent to participate in this study.

## Author Contributions

SK designed the work, collected and interpreted the data, and wrote the initial draft of the manuscript. TMima contributed to interpretation of data and assisted in the preparation of the manuscript. All other authors have contributed to data collection and interpretation and critically reviewed the manuscript. All authors approved the final version of the manuscript.

## Conflict of Interest

The authors declare that the research was conducted in the absence of any commercial or financial relationships that could be construed as a potential conflict of interest.

## Publisher’s Note

All claims expressed in this article are solely those of the authors and do not necessarily represent those of their affiliated organizations, or those of the publisher, the editors and the reviewers. Any product that may be evaluated in this article, or claim that may be made by its manufacturer, is not guaranteed or endorsed by the publisher.
